# Inertial Microfluidics-Based Separation of Microalgae Using a Contraction–Expansion Array Microchannel

**DOI:** 10.3390/mi12010097

**Published:** 2021-01-19

**Authors:** Ga-Yeong Kim, Jaejung Son, Jong-In Han, Je-Kyun Park

**Affiliations:** 1 Department of Civil and Environmental Engineering, Korea Advanced Institute of Science and Technology (KAIST), 291 Daehak-ro, Yuseong-gu, Daejeon 34141, Korea; kgy003@kaist.ac.kr; 2 Department of Bio and Brain Engineering, Korea Advanced Institute of Science and Technology (KAIST), 291 Daehak-ro, Yuseong-gu, Daejeon 34141, Korea; sonjj7@kaist.ac.kr

**Keywords:** cell sorting, *Chlorella vulgaris*, *Haematococcus pluvialis*, inertial microfluidics, microalgae isolation, microalgae separation

## Abstract

Microalgae separation technology is essential for both executing laboratory-based fundamental studies and ensuring the quality of the final algal products. However, the conventional microalgae separation technology of micropipetting requires highly skilled operators and several months of repeated separation to obtain a microalgal single strain. This study therefore aimed at utilizing microfluidic cell sorting technology for the simple and effective separation of microalgae. Microalgae are characterized by their various morphologies with a wide range of sizes. In this study, a contraction–expansion array microchannel, which utilizes these unique properties of microalgae, was specifically employed for the size-based separation of microalgae. At Reynolds number of 9, two model algal cells, *Chlorella vulgaris* (*C. vulgaris*) and *Haematococcus pluvialis* (*H. pluvialis*), were successfully separated without showing any sign of cell damage, yielding a purity of 97.9% for *C. vulgaris* and 94.9% for *H. pluvialis*. The result supported that the inertia-based separation technology could be a powerful alternative to the labor-intensive and time-consuming conventional microalgae separation technologies.

## 1. Introduction

Microalgae can mitigate carbon dioxide through photosynthesis, producing source materials for a variety of value-added products, such as biofuels, cosmeceuticals, nutraceuticals, functional foods, and commodity chemicals [1]. These microalgae are ubiquitous in environments, and they mostly exist as consortia with great dynamics and complexity in their natural habitats [2]. However, only a few special species have the aforementioned industrial potential, and it is usually realized by cultivating a single algal strain with a high content of a target product. It is therefore fundamental to isolate microalgal strains with desired features from natural environments for the sake of both laboratory investigations and successful commercial applications [3]. Another equally critical issue, when it comes to mass cultivation, is purity control. Microalgae are vulnerable to contamination by invading bacteria, protozoa, or other unwanted microalgae species, and these unplanned-yet-happened-to-be-co-inhabitants typically end up lowering biomass productivity and deteriorating the quality of the final products. It is therefore important to monitor its incursion and to remove contaminants.

Conventional methods of isolating and separating microalgae include streaking, micro-pipetting, and flow cytometry. Streaking is to spread potentially-algae-containing samples on an agar plate and then to pick a morphologically distinct colony. Although this age-old technology is still regarded as a method of choice for bacteria or fungi, it is not effective for microalgae because algal cells develop similar green colonies that are rarely distinguishable. In addition, micro-pipetting, an advanced technique of picking a single cell with a micropipette or glass capillary under microscopic observation, requires highly skilled operators [4]. It is also notoriously labor-intensive, and the sharp tip causes shear stress, potentially damaging the cells. Flow cytometer equipped with a cell-sorting module can be an alternative, but its high cost and frequent cell damage due to exposure to optical, electrical, or mechanical perturbation are still the issues to be solved [5]. As a solution to all these, therefore, this study aimed to develop a microfluidics-based microalgae separation technique, which can offer cheap operation, high throughput capability, and the production of undamaged cells.

To date, diverse microfluidic platforms have been proposed in connection with microalgal biotechnology applications: e.g., pixel-based photo-bioreactor [6], on-chip hydrothermal liquefaction [7], toxicity screening [8], and bio-solar cell [9]. Besides, attempts to separate microalgae using this microfluidic technology are also recently being made [10,11]. For example, Wang et al. designed a two-stage microfluidic chip, which could separate the microalgae by their sizes and dielectric properties [10], and Park et al. presented an acoustofluidic platform, which could obtain a target microalgal species from a heterogeneous population of cells [11]. Although these previous studies were able to achieve the separation of microalgae, their dependence on external force fields limits the accessibility of the end-users without expertise in chip technology. Therefore, this study focused on developing a passive and simple microalgae separation device so that the end-users with diverse backgrounds, especially those working on the microalgal biorefinery, can more easily take advantage of the microfluidic technologies with minimum equipment.

**Figure 1:**
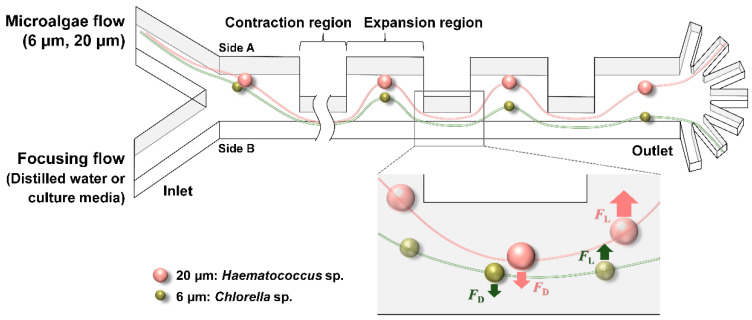
Schematic of a Contraction–Expansion Array (CEA) microchannel for the size-based separation of microalgae (*F*_D_ = Dean drag force, *F*_L_ = inertial lift force).

In this regard, among various microfluidic techniques, this study adopted inertial microfluidics, in which randomly distributed particles are aligned to specific equilibrium positions according to their sizes by the inertia of the fluid [12,13,14]. Yuan et al. recently utilized inertial microfluidics for the sheathless separation of microalgae from bacteria [15]. In contrast to their study, however, this study targeted only the separation between the microalgae. To this end, a contraction–expansion array (CEA) microchannel, which is composed of a series of regions whose width alternately contracts and expands, was particularly employed in this study (Figure 1). This kind of microchannel has been previously demonstrated as a high-throughput inertial separator in many biomedical applications [16,17,18].

In a CEA microchannel, particles are separated by the force balance between inertial lift force ($F_{L})$ and Dean drag force ($F_{D}$). The first component, the inertial lift force is affected by two sub-forces: shear-induced lift force ($F_{\mathrm{LS}}$) and wall-induced lift force ($F_{\mathrm{LW}}$). Inside the channel, particles near the channel centerline are pushed towards the channel wall by $F_{\mathrm{LS}}$, which arises from the parabolic velocity profile of the fluid, but $F_{\mathrm{LW}}$, which is generated by the pressure gradient near the wall, propels the particles back towards the channel centerline. The resultant force of these two forces is $F_{L}$, and it can be expressed as:

(1) $$F_{L}=\frac{f_{L}\rho U^{2}a^{4}}{H^{2}}$$

where $f_{L}$, ρ, U, a, and H are the lift coefficient, density of the fluid, average flow velocity, diameter of the particle, and characteristic channel dimension, respectively [19]. This $F_{L}$ causes the particles to migrate to side A in the CEA microchannel. In addition, when particles enter the contraction region from the expansion region, they experience the same effect as flowing through a curved structure, and this induces the second component, Dean drag force:

(2) $$F_{D}=3\pi \mu U_{\mathrm{VW}}a$$

where μ and $U_{\mathrm{VW}}$ are the dynamic viscosity and transverse velocity of secondary flow, respectively [19]. This $F_{D}$, formed by a radial pressure gradient due to the centrifugal force, makes the particles move to side B. Consequently, the particles are focused at the point where these two forces, $F_{L}$ and $F_{D}$, are balanced. Because the particle size has a much greater effect on the inertial lift force than the Dean drag force ($F_{L}\propto a^{4},\;F_{D}\propto a$), the relatively larger particles migrate to side A faster than the smaller ones do. Therefore, the larger the particles are, the closer to side A they are aligned, enabling size-based separation.

In this study, the same working principle was applied for the purpose of separating two microalgal species of *Chlorella vulgaris* (*C. vulgaris*) and *Haematococcus pluvialis* (*H. pluvialis*). These two freshwater species have distinct cell sizes, and they are both commercially important: *C. vulgaris* as a source of biodiesel and food supplements and *H. pluvialis* for astaxanthin production. In a CEA microchannel, we first demonstrated the inertial separation of particles by using similar-sized fluorescent microbeads under various Reynolds number (Re) conditions. The effectiveness of this technique was also then assessed for the actual algae cells in terms of the purity and reculturability of the separated algal cells.

## 2. Materials and Methods

### 2.1. Design and Fabrication of Contraction–Expansion Array (CEA) Microchannel

The CEA microchannel was designed to have seven rectangular arrays, each of which had a 1200 μm-long, 100 μm-wide contraction region and a 700 μm-long, 450 μm-wide expansion region with 80 μm of channel height, and two inlets for each of sample and focusing flow were prepared. In addition, compared to our previous study using bifurcated outlets [16], this study provided hepta-furcated outlets with larger spreading angles right after the last expansion region for more effective separation and further extended applications.

This microchannel was fabricated in poly(dimethylsiloxane) (PDMS) using conventional soft lithography techniques. A degassed mixture of PDMS prepolymer and its curing agent (Sylgard 184; Dow Corning, MI, USA) in a ratio of 9:1 was poured onto the trichloro(1H,1H,2H,2H-perfluorooctyl)silane-treated SU-8 photoresist molds and cured for 1 h on a hot plate at 80 °C. This PDMS replica was then irreversibly bonded with a glass slide by treating both with an oxygen plasma cleaner (CUTE; Femto Science, Inc., Hwaseong, Korea) for 1 min.

### 2.2. Fluorescent Microbeads and Microalgae Sample Preparation

Two kinds (6 μm red and 20 μm green) of fluorescent microbeads (Fluoresbrite Microspheres; Polysciences, PA, USA) served as surrogate particles to mimic the sorting behavior of *C. vulgaris* and *H. pluvialis* cells, respectively. These microbeads were separately prepared in 0.2% Pluronic F-127 solution with a concentration of 10^6^ particles mL^−1^ for the 6 μm beads and 10^5^ particles mL^−1^ for the 20 μm beads. The pluronic solution was used to minimize the adhesion of microbeads to the channel wall and other beads.

For algae samples, *C. vulgaris* (UTEX 265) was cultivated in a culture flask containing 50 mL of BG 11 medium [20] with operational conditions of 28 °C, the light intensity of 100 μmol photons m^−2^ s^−1^, shaking at 160 rpm, and with air supplementation through a vent cap. The second species, *H. pluvialis* (LIMS-PS-1354), which was provided by the courtesy of the Korea Institute of Ocean Science & Technology (KIOST), was cultivated in a baffled culture flask containing 200 mL of the JM medium (Culture Collection of Algae and Protozoa) under the same culture conditions, except that 2% (*v*/*v*) CO_2_ was supplied with 0.2 vvm in order to obtain high biomass productivity.

**Figure 2:**
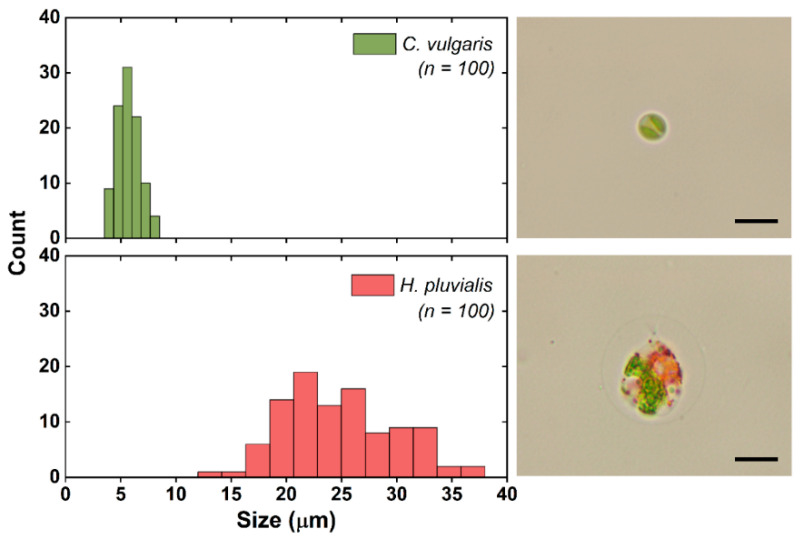
The size distribution of *C. vulgaris* and *H. pluvialis* and their respective microscopic images. Scale bar = 10 μm.

Figure 2 shows the size distributions of the two algal cells, indicating an average diameter of 5.7 μm for *C. vulgaris* and 24.8 μm for *H. pluvialis*. The size distributions of the microalgae were obtained from microscopic image analysis using ImageJ (https://imagej.nih.gov/ij/) software. Particularly, the size of *H. pluvialis* was measured including its extracellular matrix. In addition, the cell concentrations of the cultured microalgae were determined using a cell counter (LUNA-II Automated Cell Counter; Logos Biosystems, Anyang, Korea) at the exponential growth phase, and a mixed algal sample was prepared in such a way that each algal sample had a cell concentration of 10^6^ cells mL^−1^.

### 2.3. Experimental Setup and Data Analysis

Fluorescent microbeads suspension and distilled water were injected into each inlet of the CEA microchannel using syringe pumps (KDS200; KD Scientific Inc., Holliston, MA, USA), while constantly maintaining the ratio between the sample and focusing flow at 1:9. Total flow rates of 4.9, 7.4, and 9.8 mL h^−1^ were tested, corresponding to Re of 6, 9, and 12, respectively. The trajectories of the fluorescent microbeads were visualized by an inverted fluorescence microscope (IX51; Olympus, Tokyo, Japan) equipped with a charge-coupled device (DP72; Olympus).

For recording the separation of algal samples, the CEA microchannel was placed on an inverted microscope (Eclipse TS100; Nikon, Tokyo, Japan) connected with a high-speed camera (HotShot 512 sc; nac Image Technology, Simi Valley, CA, USA). The mixed algae culture solution and distilled water were injected into the channel in the same manner, but more various flow conditions (Re of 1, 3, 6, 9, 12, and 15) were examined with the total flow rates of 0.8, 2.5, 4.9, 7.4, 9.8, and 12.3 mL h^−1^, respectively.

To observe the separation of *C. vulgaris* and *H. pluvialis* in the CEA microchannel, their lateral positions at the last expansion region were captured by using a high-speed camera with frame rates of 4000–5000 fps. The images were then analyzed with ImageJ software to estimate the distance of algal cells from the channel wall of side A, and the distribution of their lateral positions was represented with a split violin plot. In addition, the outlets through which the algae cells exited (among the seven outlets) were also recorded and counted.

### 2.4. Purity and Reculturability of the Collected Algal Cells

Samples from each of the seven outlets were collected at Re of 9, and the number of cells of each microalga was measured by a cell counter (LUNA-II Automated Cell Counter; Logos Biosystems). In particular, *C. vulgaris* was found to be mainly focused to outlet 6 and *H. pluvialis* to outlet 2. As a means of quantifying the degree of cell separation, purity was defined as the percentage of specific algal cells among the total number of algal cells recovered.

(3) $$\mathrm{Purity}\;\mathrm{of}\;C.\;vulgaris\;\left(\%\right)=\;\frac{C.vulgaris_{outlet\;6}}{C.\;vulgaris_{outlet\;6}+\;H.\;pluvialis_{outlet\;6}}\;\times 100$$

(4) $$\mathrm{Purity}\;\mathrm{of}\;H.pluvialis\;\left(\%\right)=\;\frac{H.pluvialis_{outlet\;2}}{C.\;vulgaris_{outlet\;2}+\;H.\;pluvialis_{outlet\;2}}\;\times 100$$

Since this CEA microchannel-based separation is only valid when the separated algal cells are reculturable for subsequent use, the reculturability of the separated microalgae was examined by cultivating them in a 96-well plate sealed with an air-permeable membrane (Breathe-Easy; Diversified Biotech, Boston, MA, USA). Into each well, 50 μL of collected algal cells was added, and 200 μL of BG 11 medium for *C. vulgaris* and JM medium for *H. pluvialis* was supplemented. Quintuplicate algal cell samples were then cultivated for 7 d at 28 °C with 340 μmol photons m^−2^ s^−1^ of light intensity and vibration every 30 s to prevent settling.

## 3. Results and Discussion

### 3.1. Separation of Fluorescent Microbeads

**Figure 3:**
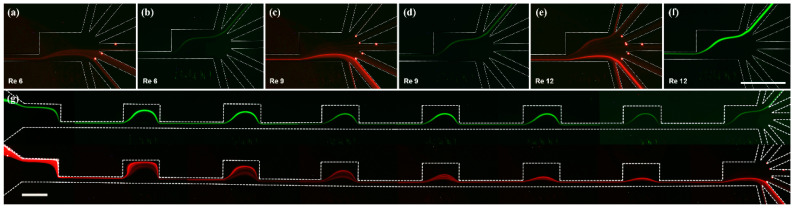
Trajectories of 6 μm (red) and 20 μm (green) fluorescent beads at the outlet of the CEA microchannel with Re of 6 (**a**,**b**), 9 (**c**,**d**), and 12 (**e**,**f**). Fluorescent microscope images of 6 μm (red) and 20 μm (green) fluorescent beads throughout the CEA microchannel at Re of 9 (**g**). Scale bar = 500 μm.

Prior to the separation of the actual algal samples, the inertial migration behavior of particles in the CEA microchannel was first characterized using similar-sized fluorescent microbeads, with Re varied from 6 to 12 (Figure 3). The smaller beads (6 μm) were found to migrate towards outlets 4–6 at Re of 6 (Figure 3a), being further pushed towards outlets 5–7 as Re was increased to 9 (Figure 3c). When the flow rate was elevated to Re of 12, however, they became dispersed in the channel rather than being focused and exited through all seven outlets (Figure 3e). In contrast, the larger beads (20 μm) appeared to be focused to outlet 2 regardless of Re (Figure 3b, d, and f). Taken together, it was found that Re of 9 was the best flow condition for the size-based separation of the beads with two different sizes of 6 and 20 μm.

At Re of 9, the trajectories of microbeads throughout the CEA microchannel were also investigated (Figure 3g). The 20 μm green beads maintained their injected initial streamline towards the upper outlet 2, whereas the 6 μm red ones gradually migrated from their injected initial position towards the bottom outlets as they passed through the contraction regions, allowing for clear separation.

### 3.2. Separation of Microalgae

**Figure 4:**
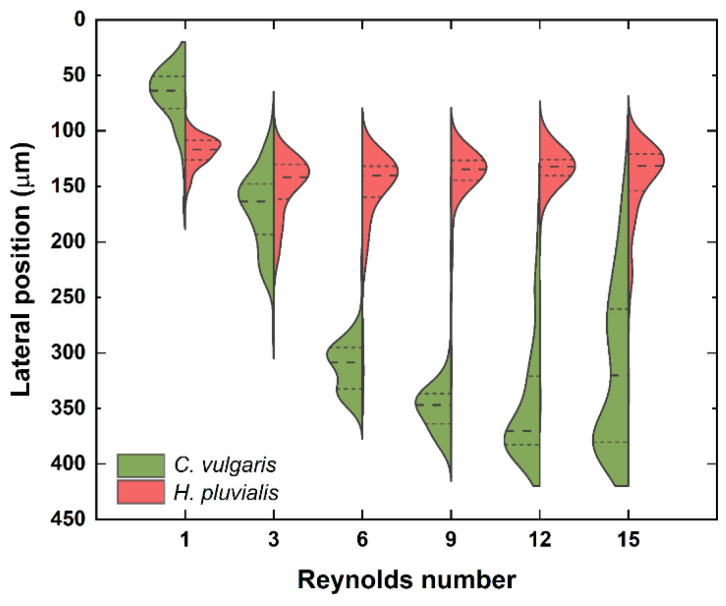
The lateral positions of *C. vulgaris* and *H. pluvialis* with Re ranging from 1 to 15 (*n* = 100 for each alga). The dashed line and short dashed line stand for median and percentiles (25th and 75th), respectively.

Based on the optimum flow rates obtained using the fluorescent microbeads, the same separation was attempted with a mixed microalgae sample (*C. vulgaris* with an average diameter of 5.7 μm and *H. pluvialis* with 24.8 μm). After passing through the seven contraction regions, *H. pluvialis* was found to have median lateral positions of 116.8–141.8 μm at all the tested Re from 1 to 15 (Figure 4). On the other hand, the median lateral positions of *C. vulgaris* increased from 63.8 μm to 346.9 μm as Re was increased from 1 to 9. In the CEA microchannel, the magnitude of the inertial lift force and Dean drag force depends on the particle size, and the inertial lift force increases more rapidly than the Dean drag force as the particle size increases due to its biquadratic dependence on the particle size ($F_{L}\propto a^{4},F_{D}\propto a$) [18]. From this principle, the larger *H. pluvialis* cells occupied their equilibrium positions near the upper outlets, being influenced mostly by the inertial lift force ($F_{L}>F_{D})$, while the smaller *C. vulgaris* cells migrated to the opposite direction, being dominated by the secondary flow-induced Dean drag force ($F_{L}<F_{D}).$ Conclusively, the two cells were most effectively separated at Re of 9 with 134.6 μm of median lateral position for *H. pluvialis* and 346.9 μm for *C. vulgaris*.

Previously, Schaap et al. have also attempted the separation of *C. vulgaris* using a spiral microchannel [21]. However, *C. vulgaris* with an average diameter of 6.0 μm was not focused but remained spread across the channel at all flow rates tested, and only the larger *Cyanothece aeruginosa* and *Monoraphidium griffithii* were sorted with a separation efficiency of 77%. This study is meaningful in that smaller *C. vulgaris* was also separately focused.

When the Re was further increased to 12, however, *C. vulgaris* also began to approach the upper outlets because the inertial lift force pushing *C. vulgaris* towards side A was enhanced by the elevated flow rate [22]. At Re of 12 and 15, although *H. pluvialis* and *C. vulgaris* still had distinct median lateral positions, their overall distributions overlapped, resulting in poor separation.

**Figure 5:**
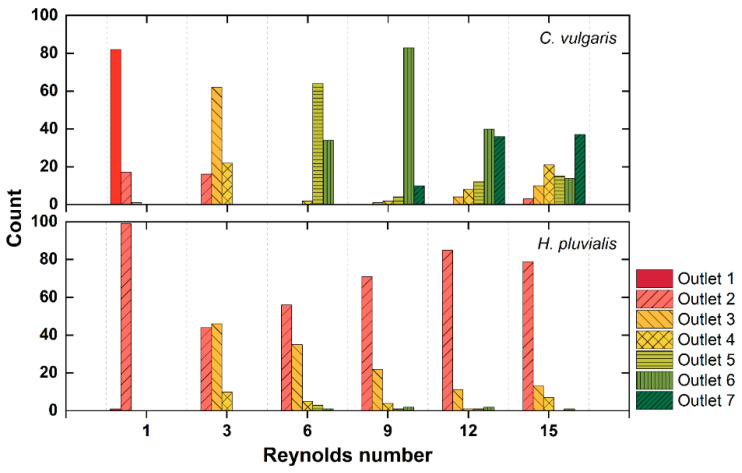
The number of *C. vulgaris* and *H. pluvialis* counted at each of seven outlets with Re ranging from 1 to 15 (*n* = 100 for each alga).

The outlet through which each algal cell exited was also monitored, and the most distinct separation was found to be achieved at Re of 9, consistent with the tendency of previous lateral position results. After passing through the CEA microchannel at Re of 9, most of the *C. vulgaris* cells were collected at outlet 6 and the *H. pluvialis* cells at outlet 2 (Figure 5). Cells of the two algal species were also separated at Re of 6 and 12, but Re of 9 was determined to be the optimal condition considering that the largest number of algal cells was collected at Re of 9. All these results were in agreement with the results from the test using fluorescent microbeads.

### 3.3. Purity and Reculturability of the Collected Algal Cells

In addition to visual confirmation using a high-speed camera, the performance of the CEA microchannel was evaluated in terms of purity and reculturability of the separated cells, which are true and practical ways of verifying its efficacy. To this end, samples from each of outlet 2 and outlet 6 were collected under the optimum operational condition of Re of 9, and their purities were calculated. As a result, it was found that the purity of *H. pluvialis* recovered from outlet 2 was 94.9%, and that of *C. vulgaris* from outlet 6 was 97.9%.

Moreover, high shear stress due to high operational flow rates could result in cell damage; thus, reculturability, another key performance indicator, was also examined by re-inoculating the separated algal cells into a fresh culture medium. Consequently, both *H. pluvialis* and *C. vulgaris* grew well without showing any sign of cell damage (Supporting Figure S1). Besides, although the separation itself failed to yield a pure single-cell culture (<100% of purity, 94.9% and 97.9%), the minority species appeared to remain suppressed during the cultivation. It was plausible that the number of contaminating cells was too low to become meaningful in number after the cultivation. This was in line with the findings of Syed et al. [23].

It is true that there are some limitations to current results, but these are expected to be sufficiently overcome in future applications. In this experiment, algae samples of high concentrations were used for the convenience of analysis. Therefore, it would be possible to achieve higher purity, for example, if natural water samples, which have far lower algal cell concentrations, are used. In addition, the dilution of the initial samples or multiple cycles of separation could also be employed as a strategy to improve purity.

## 4. Conclusions

This study proposed an inertial microfluidics-based microalgae separation technique that allows microalgae cells to be separated by their sizes in a simple, low-cost, and high-throughput manner. Without external force fields, algal cells were successfully separated (>94% of the purity), and the separated algal cells were found to be reculturable. In future studies, the parallelization of microchannel for multiplex separation, channel modification for the separation of more than two microalgae, or combination with a downstream optofluidic device for the automated sorting and identification is worth investigating. This technique can also be applied to secure axenic microalgal strains by eliminating smaller bacteria (0.5–5 μm) from microalgae.

## Data Availability

Data is contained within the article or Supplementary Material.

## Supplementary Materials

The following are available online at https://www.mdpi.com/2072-666X/12/1/97/s1. A microscopic image of algal cells obtained from each of (a) outlet 2 and (b) outlet 6 after 7 days of cultivation (Figure S1).
