# Supplementary material for: Inertial Microfluidics-Based Separation of Microalgae Using a Contraction–Expansion Array Microchannel
Source: Micromachines (Basel). 2021 Jan 19;12(1):97. doi: 10.3390/mi12010097 (PMC7833403; doi:10.3390/mi12010097)
Supplement: Supplementary file 1 [file micromachines-12-00097-s001.pdf]

Inertial Microfluidics-Based Separation of Microalgae Using a Contraction–Expansion Array Microchannel

Ga-Yeong Kim [1], Jaejung Son [2], Jong-In Han [1,*] and Je-Kyun Park [2,*]

[1] Department of Civil and Environmental Engineering, Korea Advanced Institute of Science and Technology (KAIST), 291 Daehak-ro, Yuseong-gu, Daejeon 34141, Republic of Korea; kgy003@kaist.ac.kr (G.-Y.K.)
[2] Department of Bio and Brain Engineering, Korea Advanced Institute of Science and Technology (KAIST), 291 Daehak-ro, Yuseong-gu, Daejeon 34141, Republic of Korea; sonjj7@kaist.ac.kr (J.S.)
* Correspondence: jihan@kaist.ac.kr (J.-I.H.); Tel:+82-42-350-3629; jekyun@kaist.ac.kr (J.-K. P.); Tel.: +82-42-350-4315

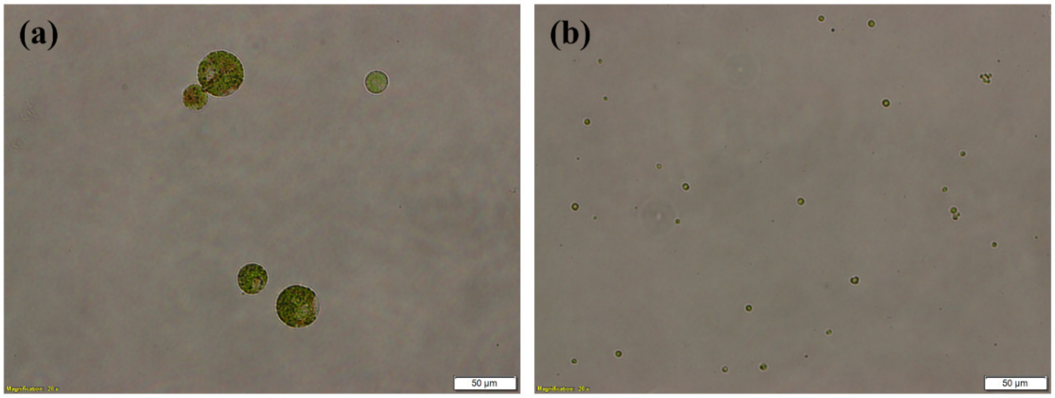

Figure S1. A microscopic image of algal cells obtained from each of (a) outlet 2 and (b) outlet 6 after 7 days of cultivation. Scale bar = 50 μm.
